# Association Between Total Serum Immunoglobulin E Levels and Persistent Pruritus Following Scabies Treatment: An Observational Study

**DOI:** 10.7759/cureus.105514

**Published:** 2026-03-19

**Authors:** Rizka Fauziyah, Agnes S Siswati, Dwi Retno A Winarni, Ichwan Ichwan, Fajar Waskito, Niken Trisnowati, Hardyanto Soebono

**Affiliations:** 1 Dermatovenereology, Faculty of Medicine, Public Health, and Nursing, Gadjah Mada University, Yogyakarta, IDN; 2 General Practice, Faculty of Medicine, Public Health, and Nursing, Gadjah Mada University, Yogyakarta, IDN

**Keywords:** immunoglobulin e, permethrin, post-scabietic itch, pruritus, scabies, serum ige

## Abstract

Background: Scabies is a common parasitic skin infestation associated with significant immune activation. Elevated total serum immunoglobulin E (IgE) levels have been reported in scabies; however, their association with persistent pruritus following treatment remains unclear.

Methods: This observational analytical study with follow-up assessment included 66 subjects (30 scabies, 36 non-scabies) recruited from Sardjito General Hospital, affiliated hospitals (Sleman Regional General Hospital), and boarding schools in Yogyakarta, Indonesia. Scabies was diagnosed using the 2020 International Alliance for the Control of Scabies (IACS) criteria. Total serum IgE levels were measured once at baseline using electrochemiluminescence immunoassay. Pruritus severity was assessed using the Visual Analogue Scale (VAS) at weeks 0, 2, 4, and 8 following treatment with topical permethrin 5% cream with a repeat application after one week according to standard treatment recommendations. Non-normally distributed data were summarized using median and interquartile range (IQR). Statistical analysis was performed using the Mann-Whitney U test and Spearman correlation.

Results: The scabies group demonstrated significantly higher total serum IgE levels compared to the non-scabies group (median 570.80 IU/mL (IQR 100.36-5179.00) vs. 36.57 IU/mL (IQR 2.92-193.00); p < 0.001). Baseline total serum IgE levels showed a strong positive correlation with pruritus severity at week eight following treatment (ρ = 0.777; p < 0.001).

Conclusion: Total serum IgE levels were significantly higher in patients with scabies and were associated with persistent pruritus after treatment. These findings suggest a possible link between systemic immune activation and ongoing symptoms. However, due to the observational study design, causal inference cannot be made, and the lack of parasitological confirmation of mite eradication should be considered when interpreting these results.

## Introduction

Scabies is a highly prevalent parasitic skin infestation caused by *Sarcoptes scabiei* var. *hominis*, particularly affecting populations in low- and middle-income countries. Despite effective acaricidal therapy, a subset of patients continues to experience persistent pruritus following treatment, which may last for weeks to months after therapy [1].

The pathogenesis of scabies-associated pruritus involves complex interactions between the mite antigens and the host immune system. A shift toward a T helper 2 (Th2)-dominant immune response during infestation leads to increased production of immunoglobulin E (IgE), activation of mast cells, and release of pruritogenic mediators [2,3]. Although elevated IgE levels have been well documented in crusted scabies, data regarding total serum IgE levels in classic scabies and their association with persistent symptoms following treatment remain limited [2].

Persistent pruritus following standard anti-scabies treatment may substantially impair quality of life, even when therapy is administered in accordance with established guidelines [4,5]. While current management strategies primarily emphasize successful mite eradication, the immunological mechanisms that may contribute to the persistence of symptoms after treatment remain insufficiently characterized [6].

This study aimed to compare total serum IgE levels between scabies and non-scabies subjects and to evaluate the association between baseline total serum IgE levels and persistent pruritus following treatment in patients treated with topical permethrin.

## Materials and methods

Study design and participants

This study employed an observational analytical design with baseline measurement of total serum IgE and prospective follow-up assessment of pruritus severity after treatment. Participants were recruited from Sardjito General Hospital, affiliated hospitals (Sleman Regional General Hospital), and boarding schools in Yogyakarta, Indonesia, between December 2024 and June 2025, during which participant recruitment, baseline assessment, treatment, and follow-up evaluations up to week eight were completed. A total of 66 participants were enrolled, consisting of 30 patients diagnosed with scabies and 36 non-scabies controls.

Inclusion and exclusion criteria

Scabies diagnosis was established according to the 2020 International Alliance for the Control of Scabies (IACS) diagnostic criteria [1], based on standardized clinical assessment. Individuals with a history of atopic disease, immunosuppression, or clinically suspected parasitic infections identified through medical history and clinical evaluation were excluded. Screening for other parasitic infections was based on clinical history and physical examination, and routine laboratory screening for helminth infections was not performed. The non-scabies group consisted of individuals without clinical or diagnostic evidence of scabies and without inflammatory or pruritic dermatologic conditions. Controls were recruited from the same clinical and community settings as the scabies group to minimize environmental and epidemiological bias.

Treatment and follow-up

All scabies patients received topical permethrin 5% cream applied to the entire body surface from the neck down and left on for 8-12 hours before washing, with a repeat application after seven days, in accordance with standard treatment recommendations. Participants were advised on environmental decontamination measures, including washing bedding and clothing. Pruritus severity was assessed using the Visual Analogue Scale (VAS) at weeks 0, 2, 4, and 8 following treatment [7]. Persistent pruritus in this study was defined as itching that remained present during the follow-up period after completion of standard permethrin therapy, based on patient-reported symptoms. Evaluation of treatment response was based on guideline-based clinical assessment, including the absence of new burrows or active scabietic lesions. Routine parasitological confirmation of mite eradication following completion of standard anti-scabies therapy was not performed, as current international guidelines recognize clinical assessment as sufficient for post-treatment evaluation in most settings, particularly in endemic or resource-limited environments [8].

Measurement of total serum IgE

Venous blood samples were collected at baseline prior to treatment in the morning between 08:00 and 10:00 a.m. to minimize diurnal variation. Total serum IgE levels were measured using an electrochemiluminescence immunoassay (ECLIA) (Elecsys® IgE II, Roche Diagnostics, Mannheim, Germany) on a cobas® e 411 analyzer (Roche Diagnostics).

Sample size calculation

Sample size was calculated for an unpaired analytical comparison with numerical outcomes (α = 0.05, power 80%), yielding a minimum of 22 subjects per group [9]. The final sample exceeded this requirement.

Statistical analysis

Data were analyzed using IBM SPSS Statistics for Windows, Version 26 (Released 2018; IBM Corp., Armonk, New York, United States). Data distribution was assessed using the Kolmogorov-Smirnov test. Because IgE levels were not normally distributed, results are presented as median (interquartile range, or IQR). Group comparisons used the Mann-Whitney U test. Associations were assessed using Spearman correlation.

Ethical considerations

This study was approved by the Medical and Health Research Ethics Committee (MHREC), Faculty of Medicine, Public Health, and Nursing, Universitas Gadjah Mada - Dr. Sardjito General Hospital (approval no.: KE-FK-1675-EC-2024). Written informed consent was obtained from all participants.

## Results

Patients with scabies had significantly higher total serum IgE levels compared to non-scabies controls. The baseline characteristics of the study participants are summarized in Table 1. Total serum IgE levels were markedly right-skewed in both groups (Kolmogorov-Smirnov p < 0.001).

**Table 1:** Baseline characteristic of study participants Continuous non-normally distributed variables are presented as median (IQR) and analyzed using the Mann-Whitney U test (*). Categorical variables were analyzed using the chi-square test (**). A p-value < 0.05 was considered statistically significant. IgE: immunoglobulin E; IQR: interquartile range; SD: standard deviation

Characteristic	Scabies (n = 30)	Non-scabies (n = 36)	p-value
Age (years, mean ± SD)	23.4 ± 6.2	22.8 ± 5.7	0.68*
Sex (male/female)	17/13	20/16	0.91**
Total serum IgE (IU/mL), median (IQR)	570.80 (100.36-5179.00)	36.57 (2.92-193.00)	<0.001*

**Figure 1:**
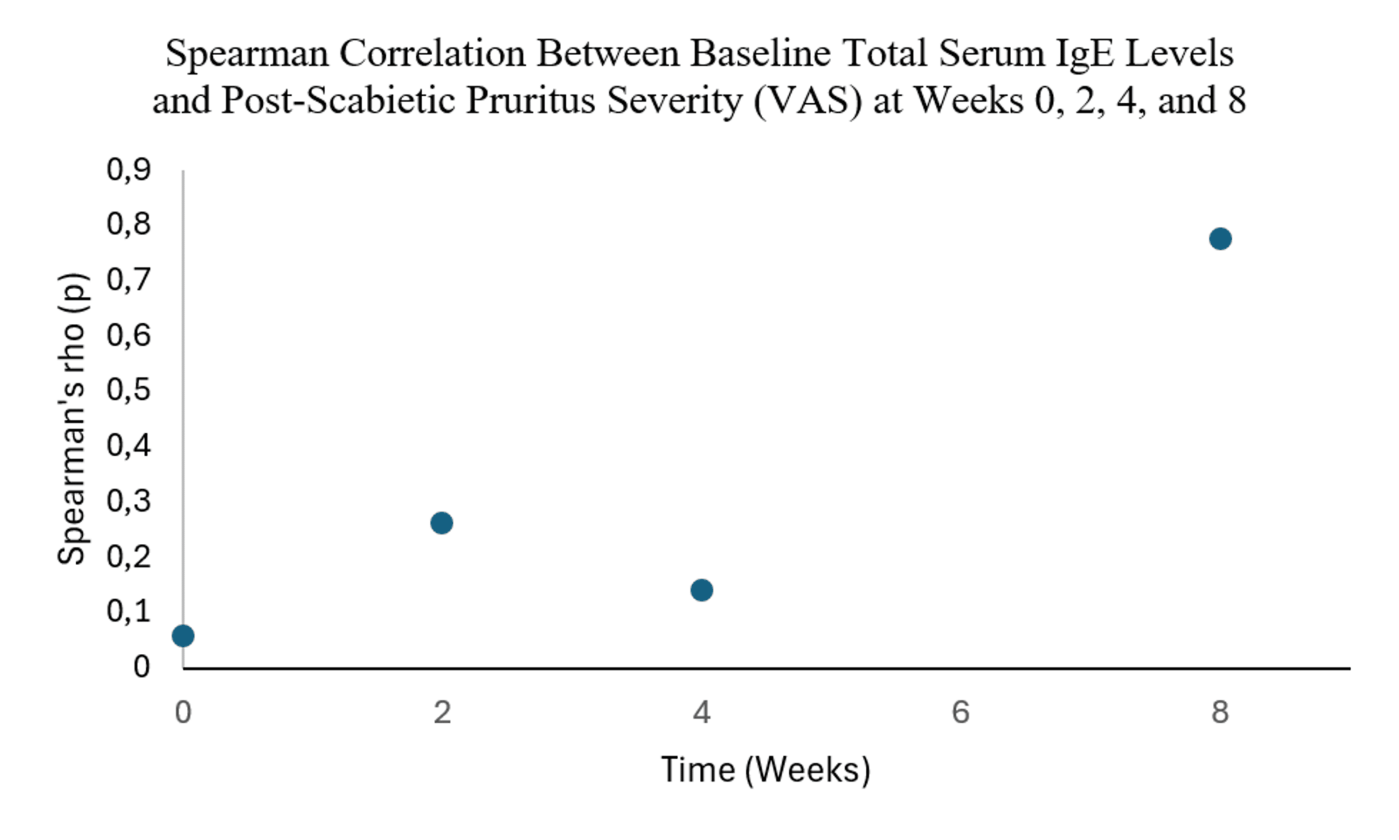
Spearman correlation between baseline total serum IgE levels measured at diagnosis and persistent pruritus severity assessed using the VAS at weeks 0, 2, 4, and 8 after treatment IgE: immunoglobulin E; VAS: Visual Analog Scale

Furthermore, baseline total serum IgE levels showed a strong positive correlation with pruritus severity at week eight following treatment with topical permethrin (ρ = 0.777; p < 0.001), as illustrated in Figure 1.

## Discussion

This study demonstrates that total serum IgE levels are significantly elevated in patients with scabies compared to non-scabies controls and are strongly associated with the severity of persistent pruritus following treatment with topical permethrin. These findings suggest that systemic immune activation may be related to ongoing symptoms after therapy.

The observed elevation of total serum IgE in scabies patients is consistent with previous studies reporting heightened humoral immune responses during *S. scabiei* infestation. Walton et al. and Bhat et al. described a predominant Th2 immune shift in scabies, characterized by increased interleukin-4, interleukin-5, and interleukin-13 production, leading to enhanced IgE synthesis and mast cell activation [2,3]. Similar elevations of serum IgE levels have been reported in both classic and crusted scabies, although the magnitude is typically greater in crusted forms due to higher parasite burden and prolonged immune stimulation [3,10,11].

Unlike earlier studies that primarily focused on crusted scabies or *S. scabiei*-specific IgE responses, the present study examines total serum IgE levels in patients with classic scabies and demonstrates a clinically relevant association with persistent pruritus after treatment. This distinction is important, as classic scabies accounts for the majority of cases encountered in routine clinical practice. Previous studies have reported that total serum IgE levels may remain elevated even after apparent clinical resolution of infestation, reflecting ongoing systemic immune activation rather than active mite infestation alone [12,13]. Accordingly, our findings suggest that in classic (non-crusted) scabies, elevated total serum IgE may persist following standard therapy and correlate with persistent pruritic symptoms. Nevertheless, total serum IgE should be interpreted as a systemic immunological marker associated with post-treatment inflammatory activity, rather than as definitive evidence of a direct causal mechanism underlying persistent pruritus. Because parasitological confirmation of mite eradication was not performed in this study, persistent pruritus observed during follow-up may also reflect delayed inflammatory responses or, in some cases, residual infestation.

A notable strength of this study is the exclusion of participants with conditions known to influence serum IgE levels and pruritus. Individuals with underlying atopic diseases such as atopic dermatitis, allergic rhinitis, and asthma were excluded, as these conditions are well-recognized causes of elevated serum IgE and chronic itch that could confound the interpretation of immunological findings. In addition, participants with a history of parasitic infections or clinical features suggestive of helminthic infestation were excluded based on detailed history taking and clinical evaluation. By applying these exclusion criteria, this study strengthens the interpretation that the observed elevation in total serum IgE is more likely related to scabies-associated immune activation rather than pre-existing allergic or parasitic conditions. This methodological approach is consistent with prior parasitological and immunological studies highlighting the importance of controlling for atopy and parasitic co-infections when interpreting IgE responses in ectoparasitic infestations [2,3,14].

The strong positive correlation between baseline total serum IgE levels and pruritus severity observed during follow-up is clinically relevant. IgE bound to high-affinity Fc epsilon RI (FcεRI) receptors on mast cells and basophils may persist for extended periods, and continued activation of these effector cells may contribute to the release of pruritogenic mediators such as histamine, leukotrienes, prostaglandins, and cytokines [2,3,15]. Similar IgE-mediated mechanisms have been implicated in other chronic pruritic conditions, supporting the biological plausibility of the observed association [16]. However, mechanistic mediators were not directly measured in this study, and causality cannot be established.

The timing of itch assessment at eight weeks following treatment is consistent with previous reports describing delayed resolution of pruritus after scabies therapy. Prior studies have documented prolonged or recurrent pruritus following treatment, suggesting that immune dysregulation may persist beyond the acute phase of infestation [1,5]. Longitudinal observations in endemic populations have similarly demonstrated delayed resolution of symptoms after therapy [17]. Our findings extend these observations by identifying baseline total serum IgE as a potential marker associated with the severity of persistent pruritus during follow-up.

The use of median and IQR to summarize total serum IgE levels reflects the highly skewed distribution observed in this study, which is consistent with the biological variability commonly reported in immunoglobulin measurements. This statistical approach provides a more accurate representation of central tendency for non-normally distributed immunological data.

From a clinical perspective, these results highlight the potential value of incorporating immunological considerations into the management of persistent pruritus following treatment. While current guidelines emphasize eradication of the mite as the primary treatment goal, adjunctive therapies targeting inflammation and itch, such as antihistamines or short-term topical corticosteroids, may be beneficial in patients with marked IgE elevation [6,18]. Identifying patients at higher risk for prolonged symptoms could help clinicians tailor post-treatment follow-up and symptom management more effectively.

Scabies remains highly prevalent in overcrowded and resource-limited settings, where delayed diagnosis, reinfestation, and repeated immune stimulation are common [13]. In such populations, persistent immune activation may be more pronounced, potentially leading to prolonged symptoms and reduced quality of life. Understanding immunological markers associated with persistent pruritus following treatment is therefore especially relevant in endemic settings and may contribute to improved patient-centered management strategies [13,14].

Limitations

This study has several limitations. First, parasitological confirmation of mite eradication was not performed at follow-up, and therefore, persistent infestation or reinfestation cannot be completely excluded as a cause of ongoing pruritus. Second, although participants with known atopic disease were excluded, other causes of elevated IgE cannot be fully ruled out. In addition, exclusion of other parasitic infections relied primarily on clinical history and examination rather than laboratory screening, which may represent a potential confounding factor affecting serum IgE levels. Third, the observational design with follow-up assessment precludes causal inference. Finally, the relatively modest sample size may limit generalizability.

## Conclusions

Total serum IgE levels are significantly elevated in scabies and are associated with persistent pruritus following treatment. These findings indicate an association between systemic immune activation and ongoing symptoms, but causal mechanisms cannot be established from this observational study, and parasitological confirmation of mite eradication was not performed. Further studies with confirmed eradication and mechanistic evaluation are required.
